# Inhibition of Hyperglycemia and Hyperlipidemia by Blocking Toll-like Receptor 4: Comparison of Wild-Type and Toll-like Receptor 4 Gene Knockout Mice on Obesity and Diabetes Modeling

**DOI:** 10.3390/biology13010063

**Published:** 2024-01-22

**Authors:** Xingyu Zhao, Jiawei Zheng, Jing Wang, Bin Li, Wuyang Huang

**Affiliations:** 1 Institute of Agro-Product Processing, Jiangsu Academy of Agricultural Sciences, Nanjing 210014, China; xzhwd0675@163.com; 2 School of Food and Biological Engineering, Jiangsu University, Zhenjiang 212013, China; yx46_zjw@126.com; 3 College of Chemical Engineering, Nanjing Forestry University, Nanjing 210037, China; wjcrystal_gl@163.com; 4 College of Food Science, Shenyang Agricultural University, Shenyang 110866, China; libinsyau@163.com

**Keywords:** TLR4, glycolipid metabolism, obesity and diabetes modeling, knockout

## Abstract

**Simple Summary:**

Toll-like receptor 4 (TLR4) is a transmembrane protein with important immune activity. However, emerging evidence has shown that TLR4 also regulates glucose and lipid metabolism by an as-yet-unknown mechanism. A study of TLR4’s role in glycolipid metabolism could contribute to the prevention of metabolic syndrome, which seriously affects human health. This study investigated the change in body weight, blood glucose, and blood lipids in both male and female wild-type (WT) and TLR4 gene knockout (TLR4^−/−^) mice during the development of obesity or diabetes models. The knockout of the TLR4 gene significantly alleviated the overweight and hyperlipidemia/hypoglycemic syndromes in mice, which confirmed that TLR4 plays an important role in glycolipid metabolism. Male mice changed more than female mice, reflecting the distinct differences in the responses between sexes. The findings of this study indicate that TLR4 has potential as a novel target to prevent and treat metabolic diseases. The established models in this study would help to screen suitable TLR4 inhibitors for application in curing obesity and diabetes.

**Abstract:**

Innate immune receptor TLR4 plays an important role in glycolipid metabolism. The objective of this study is to investigate the inhibitory effects of blocking TLR4 on hyperglycemia and hyperlipidemia by comparing WT and TLR4^−/−^ mice in obesity and diabetes modeling. The knockout of the TLR4 gene could prevent weight gain induced by a high-fat diet (HFD)/high-sugar and high-fat diet (HSHFD), and the differences in the responses existed between the sexes. It extends the time required to reach the obesity criteria. However, when mice were injected with intraperitoneal streptozotocin (STZ) after being fed by HSHFD for two months, TLR4^−/−^ mice exhibited less weight loss than WT. Blocking TLR4 alleviated the changes in body weight and blood glucose, consequently reducing the efficiency of diabetes modeling, especially for male mice. Additionally, male TLR4^−/−^ obese mice exhibit lower total cholesterol (TC) and low-density lipoprotein (LDL) levels in serum and less formation of fat droplets in the liver compared to WT. On the other hand, the knockout of TLR4 significantly increased the high-density lipoprotein (HDL) of male mice. This study should provide new insights into the role of TLR4, as well as opportunities to target novel approaches to the prevention and treatment of metabolic diseases like obesity and diabetes.

## 1. Introduction

Obesity and type 2 diabetes mellitus (T2DM) are prevalent chronic diseases around the world that are significantly harmful to human well-being and cause a substantial economic burden on healthcare systems [1,2]. There is a certain connection between obesity and diabetes, since they are both involved in the disorder/imbalance of glucolipid metabolism, and obesity management can reduce the possibility of prediabetes developing into type 2 diabetes [3,4]. For people diagnosed with diabetes, impaired blood glucose homeostasis is largely caused by changes in liver function, which plays an important role in regulating blood glucose and lipid metabolism [5]. Aberrations in gluconeogenesis, glycolysis, lipogenesis, lipolysis, and glucose transport within the liver are key factors that influence individuals with glucolipid-related disorders [6,7,8]. Obesity usually results in the excessive accumulation of lipids within the liver and disrupts its metabolic function, leading to the development of non-alcoholic fatty liver disease (NAFLD) [9].

The current drugs for obesity and diabetes mostly target the glycolipid metabolism-related enzymes or neurotransmitter receptors. For people diagnosed with obesity, they need to alleviate weight gain [10], and the foundation of successful weight loss is to reduce fat accumulation in the body [11]. The clinical drugs for treating obesity include pancreatic lipase inhibitors, glucagon-like peptide 1 (GLP1) receptor agonists and dual agonists for GLP1 and glucose-dependent insulinotropic peptide (GIP) receptors, γ-aminobutyric acid (GABA) A type receptor activators, serotonin 2C receptor agonists, opioid antagonists, dopamine-norepinephrine reuptake inhibitors, etc. [12,13,14]. At present, there is no way to completely cure type 2 diabetes. Patients with diabetes commonly take medicine including sympathomimetics biguanides, insulin secretagogues and sensitizers, α-glucosidase inhibitors, sodium-glucose co-transporter-2 inhibitors, glucagon-like peptide-1 (GLP-1) receptor agonists, dipeptidyl peptidase (DPP)-4 inhibitors, etc. [15]. However, the treatments of these drugs are sometimes accompanied by certain serious side effects and bring economic burdens for patients. Therefore, it is necessary to explore new targets for treating obesity and diabetes.

Toll-like receptor 4 (TLR4), an innate immune recognition molecule playing a key role in the initiation and development of inflammation, has recently been found to be involved in the regulation of glucose and lipid metabolism [16]. Inflammation is associated with metabolic syndrome (such as obesity, diabetes, and non-alcoholic fatty liver). Inhibiting the expression of related inflammatory factors by blocking the TLR4 signaling pathway may have a preventive effect on metabolic diseases [17,18,19]. Obesity-induced inflammation is mainly activated by the TLR4 signaling pathway, which in turn activates the downstream pathway nuclear factor kappa B (NF-κB) and causes the production of pro-inflammatory factor TNF-α, further aggravating the inflammation [20,21]. Studies have demonstrated that TLR4 deficiency can alleviate inflammation and insulin resistance in adipose tissue, and inhibit liver steatosis [22]. Phospholipase (PLA2), an enzyme produced by inflammatory cells in atherosclerotic plaques, can regulate the expression levels of HDL and LDL, and promote lipid droplet adipogenesis and accumulation in adipose tissue and the liver. Activating TLR4 by LPS can induce cPLA2 activation and lipid adipogenesis [23], which also leads to a disorder in the LDL and HDL levels of the serum. Additionally, the inhibition of TLR4/NF-κB activation can block glucose production in several hepatocyte cells and further inhibit the occurrence of hyperglycemia [24]. Therefore, TLR4 might provide a new therapeutic target for the prevention and treatment of metabolic syndrome, including obesity and diabetes.

Inhibition of TLR4 alleviates the symptoms of obesity and diabetes; however, the hypoglycemic and hypolipidemic actions of TLR4 deficiency in mice with obesity and diabetes are unknown. In this study, wild-type (WT) and TLR4 gene knockout (TLR4^−/−^) mice were used for obesity and diabetes modeling. By comparing the changes in body weight, blood glucose, and blood lipid, the role of TLR4 deficiency in stabilizing the balance of glucolipid metabolism was analyzed. In addition, the differences in liver index and physiological morphology were observed. These would help to understand the underlying molecular mechanism of TLR4 in regulating glucolipid metabolism.

## 2. Materials and Methods

### 2.1. Chemical and Reagents

The normal feed, the 60% high-fat feed, and the 45% high-fat feed for mice were from Xietong Bioenginering Co., Ltd. (Nanjing, China), and their composition and detailed ingredients are listed in Tables S1 and S2. Glucose was purchased from Sinopharm (Shanghai, China). BioFroxx streptozotocin (STZ, Saiguo Biotech, Guangzhou, China) in 0.1 M citrate buffer with pH 4.2 was freshly prepared. Blood glucose strips and a blood glucose meter were purchased from Sinocare (Changsha, China). The chemicals and reagents used in this study were all analytical grades.

### 2.2. Animals and Experiments Design

The animal models utilized in the present study included C57BL/6J wide-type (WT) mice and TLR4 gene knockout (TLR4^−/−^) mice of varying genders. The WT mice and breeding TLR4^−/−^ mice were obtained from GemPharmatech Co., Ltd. (Nanjing, China), and were housed in the Experimental Animal Center of Jiangsu University, with a stable environment maintained at 25 ± 1 °C with a 12/12 h light-dark cycle. All animal experimental procedures were performed in accordance with the guidelines of the Jiangsu Academy of Agricultural Sciences Subcommittee on Research Animal Care and Use Committee. Six mice of different sexes or types were randomly chosen and fed with a normal diet as the control group, while the others were used for obesity/diabetes modeling. The achievement ratio of the obesity/diabetes model was calculated by dividing the number of mice that meet the model criteria by the total number of mice, which is displayed as a percentage.

#### 2.2.1. Obesity Modeling

Mice aged 7–8 months were fed with a 60% high-fat diet (HFD) for 12 weeks, including 39 male and 20 female WT mice, as well as 38 male and 21 female TLR4^−/−^ mice. The fasting weight (12 h fast) of all mice was measured once every two weeks. The fasting weight gain rate (FWGR) was calculated as the percentage of the increase in fasting weight compared to the initial fasting weight at week 0. Mice with FWGR > 20% were recognized as obese, reaching the model criteria, while those 10% ≤ FWGR ≤ 20% were recognized as overweight [25].

#### 2.2.2. Diabetes Modeling

Mice at the age of 2–4 months were fed with 10% sugar water and 45% high-fat diet (HSHFD) for five months, including 27 male and 27 female WT mice, as well as 63 male and 39 female TLR4^−/−^ mice. The fasting weight of mice was measured once a month. Initial blood glucose was measured before STZ injection. The mice were fasted for 12 h and injected intraperitoneally with 100 mg/kg STZ. The drinking water was changed to be without sugar, and the feed was restored to a normal diet. One week after STZ injection, blood samples were taken from the tail vein after a 12 h fast, and fasting weight and blood glucose concentration were measured. Mice with fasting blood glucose > 11.1 mmol/L were recognized as having diabetes [26]. Those mice that did not achieve the model were fed again with an HFD for one week and given STZ injections. The STZ injection was given up to five times, and each blood glucose detection was conducted one week after the STZ injection with the diet changed to a normal diet.

### 2.3. Sample Collection

Six male WT and TLR4^−/−^ mice that reached the model criterion were chosen for each of the model groups. The mice in the obesity model groups maintained the HFD feed for another four weeks, while the mice in the diabetes model groups were fed with a normal diet for four weeks. All mice in the control groups were fed with a normal diet since the beginning of modeling with the same feed time as the obesity/diabetes models. Then, all mice were fasted overnight for 12 h, and blood samples were taken from their eyeballs to prepare serum and stored at −80 °C. All mice were anesthetized and sacrificed after blood sampling. The morphology and body width of mice were recorded. The mice’s liver tissues were removed, weighed, measured in width, and stored at −80 °C for further experiments.

### 2.4. Estimation of Serum Biochemical Indexes and Liver Indexes

The levels of triglyceride (TG), total cholesterol (TC), low-density lipoprotein (LDL), and high-density lipoprotein (HDL) in the serum of all mice were measured using a Roche Modular Cedex bio HT Biochemical Analyzer from (ALIT Life Science, Co., Ltd., Shanghai, China). The liver index was calculated by the following formula:

Liver index (%) = liver weight/body weight × 100%

### 2.5. Histological Analysis of Liver

The liver samples were harvested and placed in 10% neutral formaldehyde for 24 h, then subjected to gradient alcohol dehydration and embedded with paraffin. Sliced sections (4 µm thickness) were stained with hematoxylin and eosin (H&E) staining. The histological characteristics of the liver were visualized under a Nikon Eclipse MA200 Microscope (Nikon Instruments Inc., Melville, NY, USA). Images presented are in ×100 and ×200 magnification.

### 2.6. Statistical Analysis

The data were expressed as mean ± standard deviation (SD)/standard error of the mean (SEM). The figures were generated using GraphPad Prism 8.0 (GraphPad Software, Inc., San Diego, CA, USA). A one-way analysis of variance (ANOVA) was used to compare the means of different groups with the Tukey test (SPSS 26.0 software, Inc., Chicago, IL, USA). Two-way ANOVA was used to analyze the interaction among groups and treatment. Differences were considered significant at * *p* < 0.05.

## 3. Results

### 3.1. Effect of TLR4^−/−^ on Body Weight in Mice Fed by HFD

**Figure 1:**
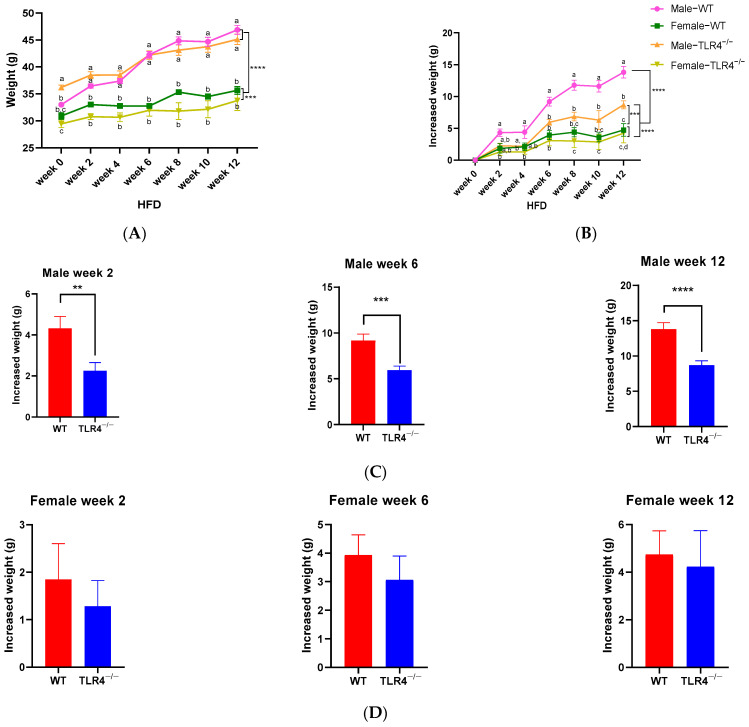
Comparison of body weight and weight gain of male/female wild-type (WT) and TLR4 gene knockout (TLR4^−/−^) mice during obesity modeling by feeding a high-fat diet (HFD) for 12 weeks. Changes in fasting body weight (**A**) and weight gain (**B**); weight gain of male (**C**) and female (**D**) mice on week 2, week 6, and week 12. Data are represented as the mean ± standard error of mean (SEM, *n* = 20–39). Different lowercase letters indicate significant differences at the same time (*p* < 0.05). Multiple groups were compared using two-way ANOVAs with Tukey’s multiple comparisons test, and differences were considered significant at ** *p* < 0.01, *** *p* < 0.001, and **** *p* < 0.0001.

During obesity modeling by feeding HFD for 12 weeks, the fasting body weight of mice all increased to some extent (Figure 1A). Compared with the WT male group, the TLR4^−/−^ male group significantly alleviated HFD-induced weight gain (*p* < 0.0001), however, the difference in weight change is not significant between female WT and TLR4^−/−^ mice (Figure 1B). The male WT mice increased body weight by about 4 g per month, with weight gain of 4.31 ± 3.51, 9.19 ± 4.18, and 13.81 ± 5.46 g at weeks 2, 6, and 12, respectively, while the weight gain of male TLR4^−/−^ mice was 2.25 ± 2.41, 5.94 ± 2.67, and 8.7 ± 3.55 g at week 2, 6, and 12, respectively, which decreased 48% (*p* < 0.01), 35% (*p* < 0.001), and 37% (*p* < 0.0001), respectively (Figure 1C). The knockout of the TLR4 gene could prevent weight gain in male mice induced by HFD. Interestingly, the mean weight gain of female mice is much lower than that of male mice, so the influence of the TLR4 knockout on body weight is not significant (*p* > 0.05, Figure 1D).

### 3.2. The Effect of TLR4^−/−^ on the Obese Modeling Rate of Mice

**Figure 2:**
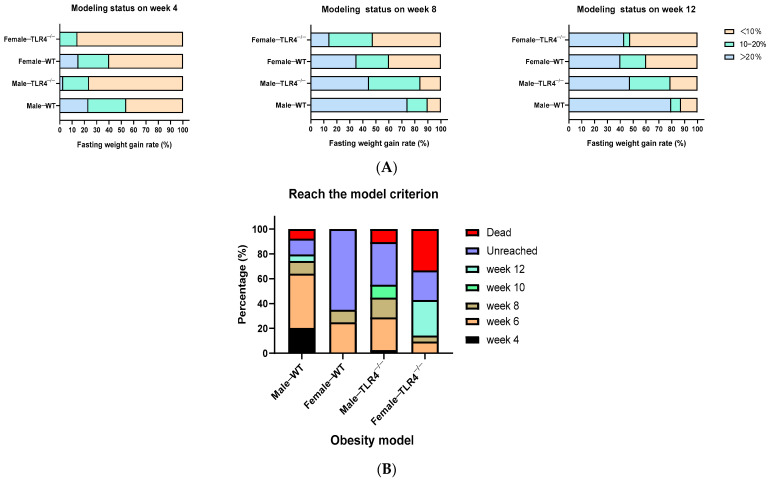
Comparison of modeling status and ratio of obesity model of male/female wild-type (WT) and TLR4 gene knockout (TLR4^−/−^) mice. (**A**) Modeling status on week 4, week 8, and week 12; (**B**) The achievement ratio of the obesity model. Fasting weight gain rate > 10% indicates overweight, while > 20% indicates obesity, reaching the model criterion.

Fed with HFD, WT mice exhibited faster weight gain than TLR4^−/−^ mice, and the body weight of males increased more than that of females. At week 4, 23% of male and 15% of female WT mice reached the obesity criterion (FWGR > 20%), and another 31% of male and 25% of female WT mice were overweight (10% ≤ FWGR ≤ 20%), respectively. However, there were no obese female TLR4^−/−^ mice, and only 3% of the male TLR4^−/−^ mice reached the obesity criterion. Some TLR4^−/−^ mice were overweight, including 21% of the males and 14% of the females. These 14% of female mice became obese at week 8, which was still the lowest obesity rate. Meanwhile, the male WT mice possessed the highest obesity rate, followed by male TLR4^−/−^ mice and female WT mice, whose rates were 74%, 45%, and 35%, respectively (Figure 2A). This also confirmed that female mice and the knockout of TLR4 were not compatible with reaching the obesity criterion. As the HFD continued to be consumed, the obese modeling speed slowed down. There were no more female WT mice reaching the obesity criterion; even the number of overweight male mice slightly decreased at week 12. The percentage of obese male TLR4^−/−^ mice was 32.12% lower than that of obese male WT mice. Moreover, the percentage of obese female TLR4^−/−^ mice increased from week 8 to week 12, which even exceeded that of obese female WT mice (Figure 2A), but accompanied by a higher proportion of mortality (Figure 2B). At the end of week 12, the final obesity modeling rates for male WT and TLR4^−/−^ mice, and female WT and TLR4^−/−^ mice, were 79%, 47%, 20%, and 43%, respectively.

### 3.3. Effects of TLR4^−/−^ on Body Width and Liver Index in Obese Mice

**Figure 3:**
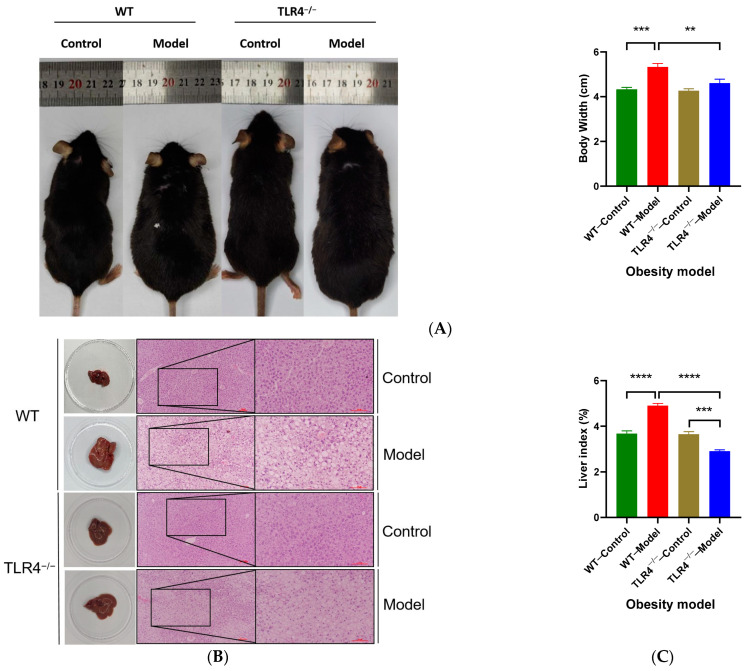

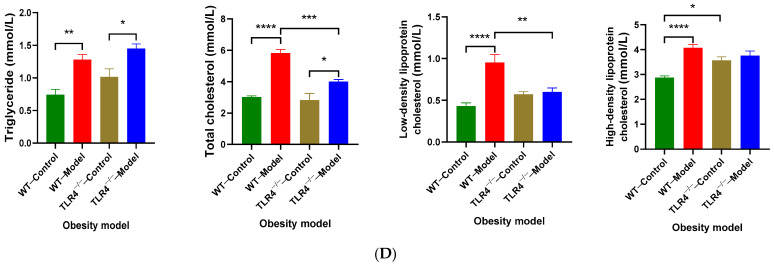
Comparison of body and blood lipids of the control and obesity model groups from male wild-type (WT) and TLR4 gene knockout (TLR4^−/−^) mice. (**A**) Body width; (**B**) liver tissue and their hematoxylin-eosin (H&E) staining images; (**C**) liver index; (**D**) triglyceride, total cholesterol, low-density lipoprotein, and high-density lipoprotein levels in serum. The control groups were fed with a normal diet (ND), while the model groups were chosen from those mice fed with a high-fat diet (HFD) that successfully reached the obesity model criterion (fasting weight gain rate > 20%). All mice continued the feed for four weeks. Data are represented as the mean ± standard error of mean (SEM, *n* = 6). One representative image from six mice in each group is shown. The scale bar represents 100 μm. Differences were considered significant at * *p* < 0.05, ** *p* < 0.01, *** *p* < 0.001, and **** *p* < 0.0001.

After obesity modeling, the six male mice that had reached an obese weight were chosen as the model groups and fed with HFD for another 4 weeks and were compared with the control groups that fed with a normal diet. The body width of the WT model group was larger than the WT control (*p* < 0.001), as well as the TLR4^−/−^ model group (*p* < 0.01) (Figure 3A; Table 1). The size of liver tissue in the WT model group was larger than that of the other groups. The H&E staining images show the accumulation of fat droplets in the liver tissue sections of both the WT and TLR4^−/−^ model groups; however, the liver fat droplets of TLR4^−/−^ model mice were significantly less than those in WT model mice (Figure 3B). Similarly, the liver index of the TLR4^−/−^ model group was lower than that of the WT model (*p* < 0.0001), and even lower than that of the TLR4^−/−^ control (*p* < 0.001, Figure 3C). There was no significant difference in serum TG levels between WT and TLR4^−/−^ model mice (*p* > 0.05). However, the TC and LDL contents of TLR4^−/−^ model mice were significantly lower than those of the WT model mice (*p* < 0.001 and *p* < 0.01, respectively). On the other hand, the TLR4^−/−^ control group had more HDL than the WT control (*p* < 0.05) (Figure 3D). These results indicated that TLR4 could play an important role in lipid metabolism, so the TLR4 gene knockout alleviated the formation of obesity.

### 3.4. Effect of TLR4^−/−^ on Body Weight and Blood Glucose in Fed by HSHFD

**Figure 4:**
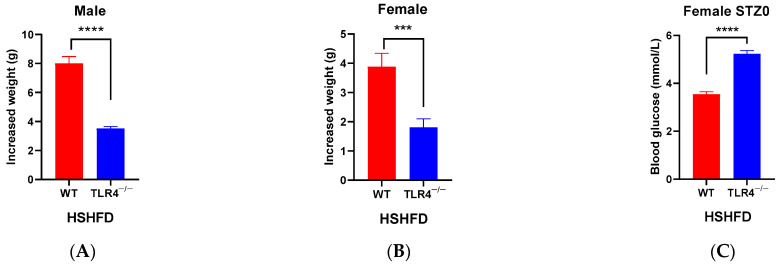

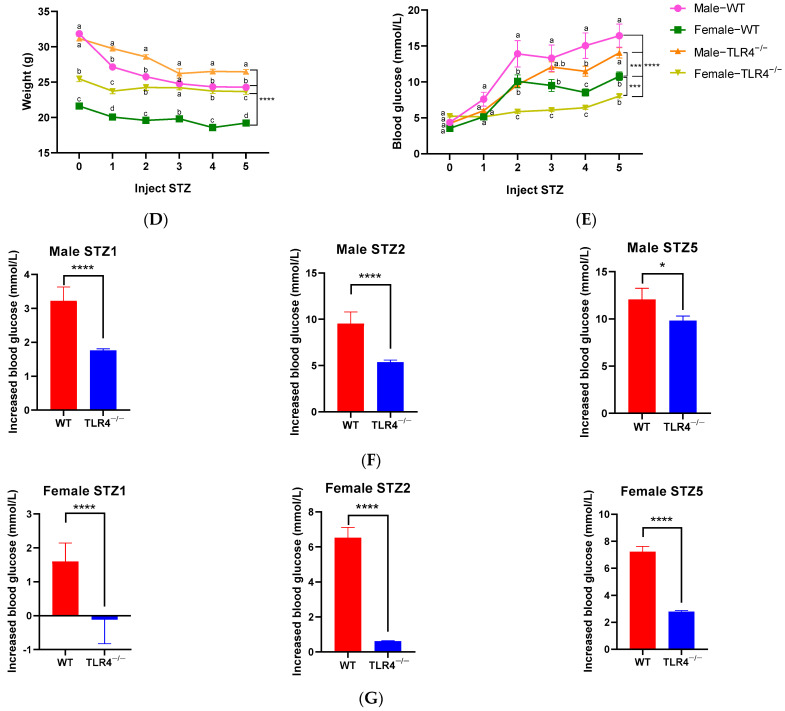
Comparison of body weight and blood glucose of male/female wild-type (WT) and TLR4 gene knockout (TLR4^−/−^) mice during type 2 diabetes modeling by feeding high-sugar and high-fat diet (HSHFD) and intraperitoneal injection of streptozotocin (STZ). Weight gain of male (**A**) and female (**B**) mice within the last two months fed with HSHFD; blood glucose of female mice before the first STZ injection (**C**); changes in fasting body weight (**D**) and blood glucose (**E**) with STZ injection; Increased blood glucose of male (**F**) and female (**G**) mice. Data are represented as the mean ± standard error of mean (SEM, *n* = 27–63). Different lowercase letters indicate significant differences with the same STZ injection (*p* < 0.05). Multiple groups were compared using two-way ANOVAs with Tukey’s multiple comparisons test, and differences were considered significant at * *p* < 0.05, *** *p* < 0.001, and **** *p* < 0.0001.

For diabetes modeling, the mice were fed with HSHFD first. All mice increased their body weight. Within the last two months, HSHFD-induced weight gain in TLR4^−/−^ male mice compared with WT male mice was largely attenuated (*p* < 0.0001, Figure 4A). The same phenomenon was observed in female mice (*p* < 0.001) (Figure 4B), but the weight gain of females was still less than the corresponding males. During the injection of STZ, the fasting body weight of all mice decreased and the blood glucose of all mice increased to varying degrees. It is worth noting that the above changes are not as obvious in female mice as in male mice, and the weight loss and blood glucose increase in male TLR4^−/−^ mice were significantly lower than that of male WT mice (*p* < 0.0001) (Figure 4D,E). By increasing the STZ injection times, blood glucose levels in mice all increased, and the increase in blood glucose in TLR4^−/−^ mice was always significantly less than that in WT mice (*p* < 0.05) (Figure 4F,G). Interestingly, the blood glucose of female TLR4^−/−^ mice decreased after the first STZ injection (Figure 4G), and they had higher initial blood glucose levels than WT mice (Figure 4C). However, there was no significant difference in initial blood glucose levels (STZ0) between WT and TLR4^−/−^ male mice (Figure S1).

### 3.5. The Effect of TLR4^−/−^ on the Diabetes Modeling Rate of Mice

**Figure 5:**
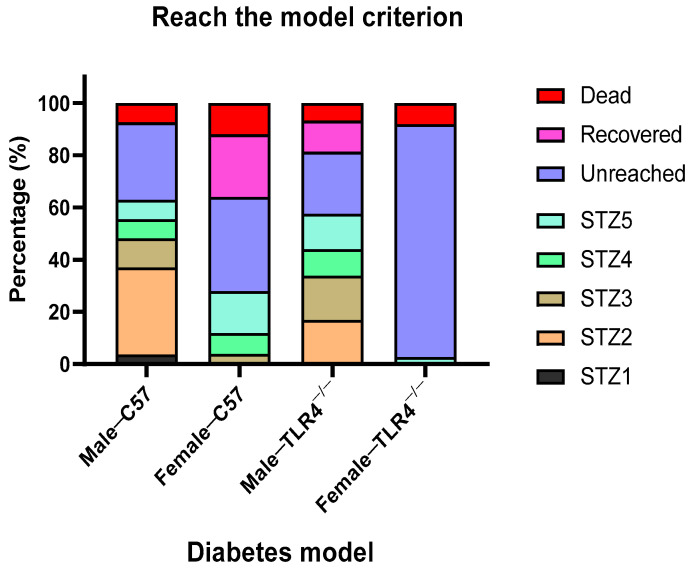
Comparison of the achievement ratio of type 2 diabetes model of male/female wild-type (WT) and TLR4 gene knockout (TLR4^−/−^) mice. Blood glucose > 11.1 mmol/L indicates diabetes, reaching the model criterion.

During the diabetes modeling process via STZ intraperitoneal injection, TLR4 gene knockout was found to postpone and alleviate the mice from reaching the criteria of type 2 diabetes, and the female mice were less prone to reaching the diabetes criterion. After the first STZ injection, only 4% of the male WT mice reached the diabetes criterion (blood glucose > 11.1 mmol/L). With two STZ injections, 37% WT and 17% TLR4^−/−^ male mice reached the diabetes criterion. At the same time, there were no female diabetic mice in both WT and TLR4^−/−^ groups. As STZ injections continued on the mice with blood glucose levels less than 11.1 mmol/L, the percentage of diabetic mice that reached the model criterion increased, except that only 3% TLR4^−/−^ female mice reached the model criterion after four STZ injections, and none reached it even with the fifth STZ injection. It is worth noting that some diabetic mice could recover their blood glucose to less than 11.1 mmol/L without further STZ injections. The final diabetic modeling rates of male WT, male TLR4^−/−^, and female WT mice were 63%, 57%, and 28%, respectively (Figure 5).

### 3.6. Effects of TLR4^−/−^ on the Body and Liver Body Width and Liver Index in Type 2 Diabetic Mice

**Figure 6:**
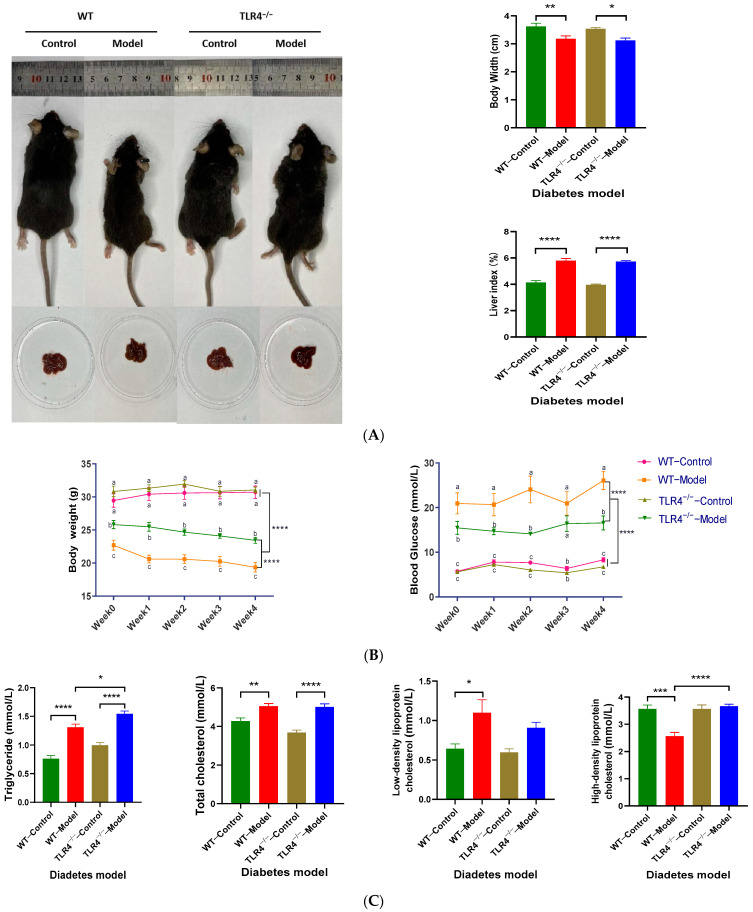
Comparison of body and blood glucose and lipids of the control and diabetes model groups from male wild-type (WT) and TLR4 gene knockout (TLR4^−/−^) mice. (**A**) Body width, Liver index, and the photos of mice and liver tissues; (**B**) changes in fasting body weight and blood glucose within four weeks; (**C**) triglyceride, total cholesterol, low-density lipoprotein, and high-density lipoprotein levels in serum. The control groups were fed with a normal diet (ND), while the model groups were chosen from those mice fed with a high-sugar and high-fat diet (HSHFD) and injected with streptozotocin (STZ) that successfully reached the diabetes model criterion (blood glucose > 11.1 mmol/L). All mice continued the feed for four weeks. Data are represented as the mean ± standard error of mean (SEM, *n* = 6). One representative image from six mice in each group is shown. Different lowercase letters (a, b, and c) indicate significant differences on the same time (*p* < 0.05). Differences were considered significant at * *p* < 0.05, ** *p* < 0.01, *** *p* < 0.001, and **** *p* < 0.0001.

The male diabetic mice chosen as the model groups were compared with the control groups after feeding with a normal diet for 4 weeks. There were significant differences in the body width and liver index between the model and control groups (*p* < 0.05), but no differences between the WT and TLR4^−/−^ groups (*p* > 0.05) (Figure 6A). However, the changes in fasting weight and blood glucose during four weeks exhibited significant differences between the WT and TLR4^−/−^ model mice (*p* < 0.05), which also confirmed the down-regulatory effect of TLR4^−/−^ on blood glucose (Figure 6B). Additionally, male TLR4^−/−^ model mice exhibit higher TG and HDL levels in serum compared to the WT (*p* < 0.05 and *p* < 0.0001, respectively), while there were no significant differences in the serum TC and LDL levels between WT and TLR4^−/−^ model mice (*p* > 0.05, Figure 6C). These findings further confirmed that TLR4 could play an important role in glucose and lipid metabolism, so TLR4 gene knockout alleviated the formation of diabetes and dyslipidemia.

## 4. Discussion

Obesity and diabetes are chronic glucolipid metabolic disorders affecting human health worldwide. Inflammatory reactions can be triggered by obesity and diabetes and an increase in adipose and blood glucose involves the innate immune system activation [27]. Moreover, insulin resistance becomes more severe as the release of pro-inflammatory/inflammatory chemokines and cytokines in the body increases [28,29,30]. The innate immune factor TLR4 is associated with inflammation and glucolipid metabolism, which is linked to insulin resistance, glycolysis, pyruvate oxidative decarboxylation, adipogenic gene expression, and intestinal permeability and flora [31,32,33,34,35]. The TLR4 activation by binding to myeloid differentiation factor 88 (MyD88) can activate the downstream (NF-κB), induce the product of pro-inflammatory/inflammatory factors, and result in glucolipid metabolism disorders, while TLR4-specific deletion can improve insulin resistance and glucose tolerance, depress the differentiation of preadipocyte, and decrease the accumulation of lipids [15,36]. This study confirmed that the knockout of the TLR4 gene significantly alleviated the overweight and hyperlipidemia/hypoglycemic syndromes in mice during the process of constructing obese/diabetes models.

The accumulation of lipids in the body is closely related to the development of obesity, which is determined by the capability of lipid metabolism [37]. Previous studies demonstrate that TLR4 is distributed in various tissues and organs, including the liver, intestine, and adipose tissue, and an increase in the expression of TLR4 was observed in the liver and adipose tissues of obese rats and mice fed with a high-fat diet [21,38,39]. The overexpression of TLR4 is associated with the accumulation of fat granules in the liver and lipid degeneration of liver cells, which has been verified in non-alcoholic steatohepatitis of mice or rats [21,39,40]. Gut microbiota *Prevotella copri* colonization could increase fat accumulation in pigs by activating TLR4 signaling pathways to increase the permeability of the intestinal barrier and cause a chronic inflammatory response in the host. Subsequently, the gene expression related to lipogenesis and fat accumulation was significantly upregulated, while the gene expression related to lipolysis and lipid transport was reduced [41]. Lipopolysaccharide and palmitic acid increased cholesterol accumulation via the activation of the TLR4/MyD88/NF-κB signaling pathway [42]. Therefore, knocking out TLR4 or the inhibition of TLR4/MyD88/NF-κB can reduce levels of total and free cholesterol. In this study, it was difficult to increase body weight in TLR4 knockout mice during HFD feeding, and the knockout of the TLR4 gene could reduce liver fat droplets and downregulate the TC and LDL levels in the serum of obese mice, which might be due to the disruption of the TLR4/MyD88/NF-κB signaling pathway, inhibiting cholesterol accumulation. However, the level of TG in serum also increased in TLR4^−/−^ obese or diabetic mice, which was consistent with the findings of Pang et al. [43] in the TLR4^−/−^ mice model after fasting. Our results confirmed the important role of TLR4 in regulating lipid metabolism and the potential hypolipidemic effect of blocking TLR4.

On the other hand, TLR4 is involved in glucose metabolism. Hyperglycemia leads to an increase in the mRNA and protein expression of TLR4 in the monocytes, and TLR4 deficiency can inhibit the high glucose-induced inflammatory response [44]. Studies have demonstrated that blocking TLR4 can improve the insulin-dependent intake of glucose, alleviating insulin resistance induced by obesity in mice [45]. It is well known that insulin resistance is closely related to the development of diabetes. Similarly, TLR4 overexpression has also been observed in type 2 diabetes patients [46]. TLR4 deficiency has a preventive effect on spontaneous autoimmune diabetes in non-obese diabetic mice [47,48]. In this study, TLR4 deficiency also prevents or delays the occurrence of type 2 diabetes in mice induced by HSHFD and STZ injection. TLR4 deficiency could lead to a decrease in fasting blood glucose in diabetic mice, which might be attributed to the decrease in the activity of pyruvate dehydrogenase complex (PDC) in skeletal muscle that promotes the circulation between glycolysis and gluconeogenesis [15,43]. The better ability of TLR4^−/−^ mice to maintain the homeostasis of body weight and blood glucose further confirmed that TLR4 can be responsible for glucose metabolism regulation and blocking TLR4 may contribute to some hypoglycemic effects.

Interestingly, there were distinct differences in glucose and lipid metabolism between male and female mice, including different weight gain during HFD/HSHFD feeding, blood glucose changes induced by STZ, and the impact of TLR4 knockout. Extensive data demonstrated that biological and psychosocial differences in gender greatly impacted the progression of disease and complications [49,50]. Elderly women had a greater ability to self-dispose of glucose than men; however, there was no significant difference in insulin secretion between them [51]. Previous studies also suggested that different hormone secretion in mice of different genders might affect energy intake in high-fat diets [52]. Contrary to the finding of Ma et al. [52] that females responded much more strongly than males for long-term bisphenol A (BPA) exposure-induced metabolic disorders, here males responded more strongly than females for diet-induced obesity and STZ-induced diabetes. The greater sensitivity of male mice to STZ was confirmed by Gurley et al. [53] during the development of the diabetes model because the pancreatic islet β-cells of males are more prone than those of females to STZ-induced cytotoxicity [54]. The strong influence of gender also exerts in BPA-induced inflammation [55]. Blocking TLR4 can improve the insulin-dependent intake of glucose and alleviate insulin resistance induced by HFD in mice, which may be because the knockout of TLR4 leads to a decrease in inflammatory factors. In addition, TLR4 deficiency in pro-opiomelanocortin (POMC) neurons can promote heat production and maintain a balance of lipid metabolism, but this ability only exists in male mice, which in turn increases the induction of obesity in female mice [56]. A slightly higher obesity rate was found in female TLR4-/- mice compared to female WT mice in this study, but nearly no female diabetic mice were obtained in the TLR4 deficiency groups. Shi et al. also reported female C57BL/6 mice lacking TLR4 had increased obesity but were partially protected against high-fat-diet-induced insulin resistance [31,56]. Females were resistant to the effects of diet and STZ in this study, so only male model mice were further studied, which may be the reason why male animals are more popular for study [54].

## 5. Conclusions

The different changes in body weight and blood glucose in WT and TLR4^−/−^ mice during the development of the obesity or diabetes models confirmed that the innate immune receptor TLR4 plays an important role in glycolipid metabolism. Obviously, TLR4 knockout alleviated these changes and reduced the modeling efficiency of high-fat diet-induced obesity or STZ-induced diabetes. Weight gain, blood glucose, blood lipid, and liver fat droplets in the TLR4-deficient mice were lower than those in WT mice, suggesting hypolipidemic and hypoglycemic effects of blocking TLR4. The greater sensitivity of male mice to dietary interference and the tolerance of female mice to STZ also caused the complicated gender differences of TLR4 influence. Taken together, the findings of this study indicate that TLR4 has potential as a novel target to prevent and treat metabolic diseases. The established models in this study would help to screen suitable TLR4 inhibitors for application in curing obesity and diabetes. Further studies to explore the detailed mechanism of the TLR4 signaling pathway in regulating glucose and lipid metabolism are still needed in the future.

## Figures and Tables

**Table 1:** Changes in fasting body weight of male wild-type (WT) and TLR4 gene knockout (TLR4^−/−^) mice.

Week	WT−Control (g)	WT−Model (g)	TLR4^−/−^−Control (g)	TLR4^−/−^−Model (g)
0	31.25 ± 1.46 ^C,D,b^	32.73 ± 2.37 ^E,b^	36.61 ± 3.03 ^A,a^	35.84 ± 1.47 ^C,a^
2	32.18 ± 1.11 ^B,C,b^	36.78 ± 2.73 ^D,a^	36.00 ± 2.84 ^A,a^	37.99 ± 1.53 ^B,C,a^
4	29.96 ± 1.61 ^D,c^	38.68 ± 2.68 ^D,a^	34.80 ± 3.38 ^A,b^	38.40 ± 2.50 ^B,C,a^
6	33.09 ± 1.34 ^A,B,C,b^	43.08 ± 3.38 ^C,a^	36.33 ± 2.97 ^A,b^	42.61 ± 3.06 ^A,B,a^
8	33.59 ± 1.59 ^A,B,b^	45.85 ± 4.10 ^B,C,a^	36.42 ± 3.33 ^A,b^	44.38 ± 4.31 ^A,a^
10	34.01 ± 1.79 ^A,B,b^	46.08 ± 5.08 ^B,a^	37.29 ± 3.28 ^A,b^	45.16 ± 4.30 ^A,a^
12	34.60 ± 2.56 ^A,B,b^	48.29 ± 4.38 ^B,a^	37.13 ± 3.07 ^A,b^	45.40 ± 4.71 ^A,a^
16	34.73 ± 1.91 ^A,c^	53.39 ± 2.85 ^A,a^	35.07 ± 2.84 ^A,a^	46.29 ± 5.76 ^A,b^

All data were expressed as mean ± standard deviation (SD, *n* = 6). Different uppercase letters (A, B, C, D, and E) indicate significant differences within the same column (*p* < 0.05). Different lowercase letters (a, b, and c) indicate significant differences within the same row (*p* < 0.05). The control groups were fed with a normal diet (ND), while the model groups were chosen from those mice fed with a high-fat diet (HFD) that successfully reached the obesity model criterion (increased weight percentage > 20%).

## Data Availability

The data presented in this study are available upon request.

## Supplementary Materials

The following supporting information can be downloaded at: https://www.mdpi.com/article/10.3390/biology13010063/s1, Figure S1: Comparison on blood glucose of male mice before the first streptozotocin (STZ) injection between wild type (WT) and TLR4 gene knockout (TLR4^−/−^) mice; Table S1: The composition of feeds; Table S2: The detail ingredients of mice high-fat feeds.
