# Supplementary material for: Inhibition of Hyperglycemia and Hyperlipidemia by Blocking Toll-like Receptor 4: Comparison of Wild-Type and Toll-like Receptor 4 Gene Knockout Mice on Obesity and Diabetes Modeling
Source: Biology (Basel). 2024 Jan 22;13(1):63. doi: 10.3390/biology13010063 (PMC10813444; doi:10.3390/biology13010063)
Supplement: Supplementary file 1 [file biology-13-00063-s001.zip › biology-2782107-supplementary.pdf]

## Supplementary Materials

**Table S1.** The composition of feeds.

| Composition | **Normal feed** | | **45% High-fat feed** | | **60% High-fat feed** | |
|---|---|---|---|---|---|---|
| | **Energy supply proportion** (calorie ratio) | **Quality proportion** | **Energy supply proportion** (calorie ratio) | **Quality proportion** | **Energy supply proportion** (calorie ratio) | **Quality proportion** |
| Fat | 10% | 4.5% | 45% | 23.6% | 60% | 35% |
| Protein | 20% | 23% | 20% | 23.7% | 20% | 26% |
| Carbohydrates (including fructose and cellulose) | 70% | 40.9% | 35% | 41.4% | 20% | 36% |

**Table S2.** The detail ingredients of mice high-fat feeds.

| | 45% High-fat feed | | 60% High-fat feed | |
|---|---|---|---|---|
| | **Energy supply / kcal** | **Quality / gm** | **Energy supply / kcal** | **Quality / gm** |
| Casein | 800 | 200 | 800 | 200 |
| L-Cytine | 12 | 3 | 12 | 3 |
| Corn Starch | 291 | 72.8 | 0 | 0 |
| Maltodextrin | 400 | 100 | 500 | 125 |
| Sucrose | 691.2 | 172.8 | 275 | 68.8 |
| Cellulose | 0 | 50 | 0 | 50 |
| Soybean oil | 225 | 25 | 225 | 25 |
| Lard | 1598 | 177.5 | 2205 | 245 |
| Mineral Mix S10026 | 0 | 10 | 0 | 10 |
| DiCalcium Phosphate | 0 | 13 | 0 | 13 |
| Calcium Carbonate | 0 | 5.5 | 0 | 5.5 |
| Potassium Citrate, 1 $H_2O$ | 0 | 16.5 | 0 | 16.5 |
| Vitamin Mix V10001 | 40 | 10 | 40 | 10 |
| Choline Bitartrate | 0 | 2 | 0 | 2 |

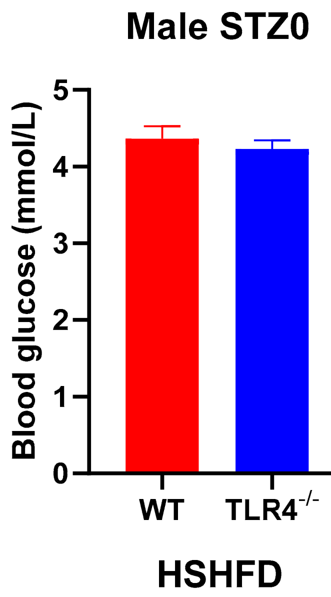


**Figure S1.** Comparison on blood glucose of male mice before the first streptozotocin (STZ) injection between wild type (WT) and TLR4 gene knockout (TLR4$^{-/-}$) mice. Data are represented as the mean ± standard error of mean (SEM, n = 27-63).
